# A supervised protein complex prediction method with network representation learning and gene ontology knowledge

**DOI:** 10.1186/s12859-022-04850-4

**Published:** 2022-07-25

**Authors:** Xiaoxu Wang, Yijia Zhang, Peixuan Zhou, Xiaoxia Liu

**Affiliations:** 1 School of Information Science and Technology, Dalian Maritime University, Dalian, 116024 Liaoning China; 2 Department of Neurology and Neurological Sciences, Stanford University, Stanford, CA 94305 USA

**Keywords:** Protein complex prediction, Supervised learning, Network representation learning, Protein–protein interaction networks

## Abstract

**Background:**

Protein complexes are essential for biologists to understand cell organization and function effectively. In recent years, predicting complexes from protein–protein interaction (PPI) networks through computational methods is one of the current research hotspots. Many methods for protein complex prediction have been proposed. However, how to use the information of known protein complexes is still a fundamental problem that needs to be solved urgently in predicting protein complexes.

**Results:**

To solve these problems, we propose a supervised learning method based on network representation learning and gene ontology knowledge, which can fully use the information of known protein complexes to predict new protein complexes. This method first constructs a weighted PPI network based on gene ontology knowledge and topology information, reducing the network's noise problem. On this basis, the topological information of known protein complexes is extracted as features, and the supervised learning model SVCC is obtained according to the feature training. At the same time, the SVCC model is used to predict candidate protein complexes from the protein interaction network. Then, we use the network representation learning method to obtain the vector representation of the protein complex and train the random forest model. Finally, we use the random forest model to classify the candidate protein complexes to obtain the final predicted protein complexes. We evaluate the performance of the proposed method on two publicly PPI data sets.

**Conclusions:**

Experimental results show that our method can effectively improve the performance of protein complex recognition compared with existing methods. In addition, we also analyze the biological significance of protein complexes predicted by our method and other methods. The results show that the protein complexes predicted by our method have high biological significance.

## Introduction

As the material basis of life, protein plays a crucial role in cell life activities and participates in almost all life activities. Proteins do not act alone but form complexes with other proteins. Therefore, predicting protein complexes is very important to comprehensively and deeply understand cell composition and life processes. Many methods exist to predict protein complexes, such as tandem affinity purification (TAP) and mass spectrometry. However, this experimental method costs a lot of human resources. Therefore, quickly and efficiently predicting protein complexes from the protein–protein interaction network has become a fundamental problem. With the rapid development of high-throughput technology, much protein–protein interaction (PPI) data [1] has been generated in recent years, which makes it possible to predict protein complexes from protein–protein networks by computational methods. PPI network constructed from PPI data can be regarded as an undirected graph, in which nodes represent proteins and edges illustrate interactions between proteins. Protein complexes usually correspond to dense sub-graphs in PPI networks.

Based on the idea of converting large-scale protein–protein interaction data into a network structure, many methods have been proposed to predict protein complexes in the PPI network. Bader et al. proposed a protein complex prediction algorithm MCODE [2], which weights the nodes according to their neighborhood density. Moreover, the node with the most significant weight is selected as the seed node. Then, the seed nodes are expanded iteratively to form protein complexes. Liu et al. proposed a protein complex prediction algorithm CMC [3] to merge the largest sub-graph. The algorithm searches the largest sub-graph from the weighted network and calculates sub-graphs weighted density. Then combines the highly overlapped sub-graphs to form protein complexes. Similar algorithms such as LCMA [4] and CFinder [5] predict protein complexes by searching and merging sub-graphs. In addition, some methods, such as COACH [6] and Core [7], have been proposed to predict protein complexes based on the core attachment junction structure. Nepuse et al. proposed a new method ClusterONE [8], to predict overlapping protein complexes. It designs a new calculation method to measure the cohesion of sub-graphs. It selects the node with a higher degree as the seed node. Then, a greedy algorithm expands the seed node to make the sub-graphs obtain higher cohesiveness until no seed node forms a protein complex. Similar methods include SE-DMTG [9] and HGCA [10], based on point expansion methods to predict protein complexes from protein interaction networks. Xu et al. proposed the CPredictor2.0 [11] algorithm, which first grouped proteins with similar functions, clustered each group using the Markov clustering algorithm, and merged overlapping protein complexes. Meng et al. proposed the DPC-HCNE [12] algorithm, which first compresses the PPI network into a smaller PPI network through heuristic hierarchical compression. Then apply the network representation learning algorithm DeepWalk [13] to construct a weighted PPI network. Finally, use the nuclear connection clustering method to predict protein complexes. Wang et al. proposed the EWCA [14] algorithm, which uses the structural similarity between nodes and their neighborhoods to determine the core. In addition, it presents a new method of predicting attachment proteins by adding them to the corresponding center to form protein complexes. Xu et al. proposed the GANE [15] algorithm, which uses the clique mining method to generate candidate cores. Then select seed cores from the candidate cores. If the degree of connection between the protein and the seed core exceeds the threshold, add the protein to the core to obtain a protein complex. The methods mentioned above are all unsupervised learning methods. They predict protein complexes based on the topological information of the protein interaction network and cannot use the data of known protein complexes.

Recently, supervised learning methods have been successfully applied in protein complex prediction, which can use the information of known protein complexes to predict new protein complexes. Yu et al. proposed the SLPC [16] method. This method first obtains the characteristics of the protein complex from the weighted and unweighted network and trains the logistic regression model. Then finds the largest sub-graph from the PPI network as the core and uses the model to add auxiliary nodes to the center to obtain protein complexes. Zhu [17] et al. proposed a semi-supervised network embedding model. It first selects the key neighborhood node as a vertex attribute and obtains the first-order approximation of the vertex. Then it designs a three-layer GCN to calculate the second-order approximation of the vertex and optimizes the first-order approximation. Finally, the model is obtained by second-order approximation and used to identify protein complexes. Faridoon [18] et al. combined the support vector machine with the ECOC algorithm. In addition, the physical properties of amino acids and various topological information are used as features to predict protein complexes from the PPI network. These methods usually extract features from known protein complexes, train a classification model based on the features. Then use the trained classification model to predict protein complexes from the protein interaction network. However, the presence of a large amount of noisy data in the PPI network. In addition to the fact that many features exist only in specific networks and are not universal, leads to uncertainty in the classification model. Therefore, obtaining effective features from known protein complexes is the key to supervised learning algorithms. In addition, the above-mentioned unsupervised learning methods and supervised learning methods are only explored in the yeast PPI network.

In this paper, we propose a protein complex prediction method based on supervised learning, which can fully use the information of known protein complexes. Moreover, to reduce the noise problem in the network and mine the biological information contained in the protein network, we introduce gene ontology (GO) knowledge [19] to construct a weighted PPI network. Furthermore, to further improve the performance of the protein complex prediction method, we use network representation learning to obtain the vector representation of the protein complex. We first use the GO knowledge to weight the PPI network and filter out the low-confidence relationship in the PPI network. Secondly, we extract the rich topological information of protein complexes as features and construct the training set based on the weighted and unweighted PPI networks. Train the supervised learning model SVCC according to the constructed training set, and use the SVCC model to predict candidate protein complexes from the PPI network. Then, we apply the network representation learning method to obtain the vector representation of each node in the PPI network and get the vector representation of the protein complex through the protein node representation. Finally, train the random forest model RF [20] according to the vector representation of the training set complex, and the candidate protein complexes are classified using the RF model. The protein complexes marked as positive examples are the final predicted protein complexes. To verify the performance of the proposed method, we conduct experiments on the yeast PPI network DIP [21] and the human PPI network HPRD [22]. Experimental results show that our method is superior to existing methods in predicting protein complexes in the PPI network. In addition, we are considering the particularity of the relationship between human proteins. We also analyzed the biological significance of the protein complexes predicted by our method and other methods. Experimental results show that our method can predict protein complexes with biological significance.

## Methods

**Fig. 1:**
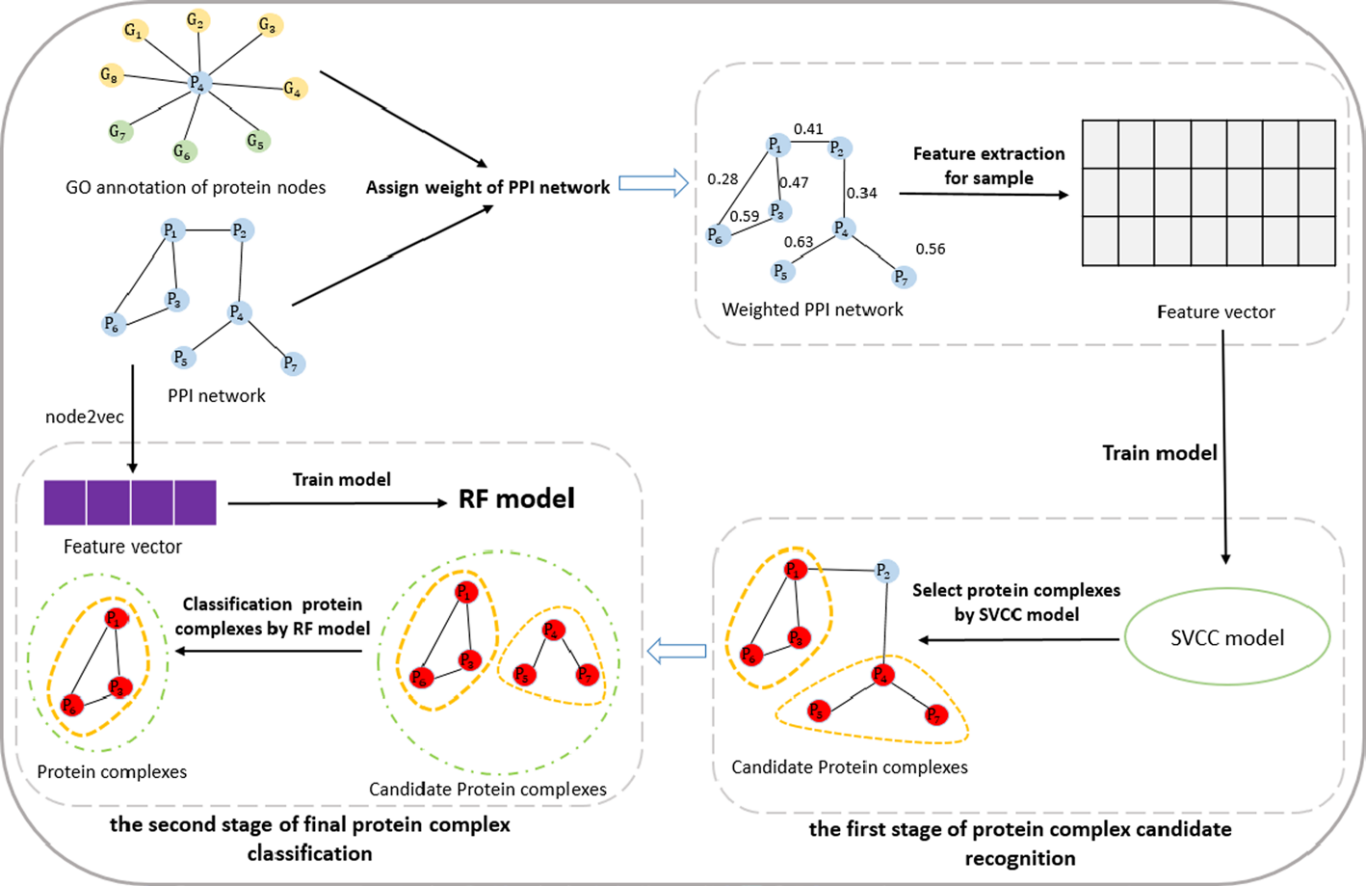
Overall flow chart of our method

We detail our protein complex prediction method in this section. Our method mainly includes four parts: (1) weighted PPI network using GO knowledge; (2) generating supervised features for protein complex prediction; (3) the first stage of protein complex candidate recognition; (4) the second stage of final protein complex classification. Figure 1 shows the overall workflow of our method.

### Weighted PPI network using GO knowledge

A protein interaction network is a basis for using computational methods to predict protein complexes. However, due to the limitations of technology and the flow characteristics of the protein interaction network, protein interaction data sets generated by high-throughput experiments often contain many a lot of noisy data [23–25]. There are two main types of noise relationships in PPI networks: false negatives and false positives. A false-negative relationship refers to an interaction relationship between two proteins that has not been discovered or documented in a database. A false positive relationship refers to the absence of an interactive relationship between two proteins, which is incorrectly recorded and stored in the protein interaction database due to experimental error. To solve this problem, researchers found that applying topological characteristics of protein–protein interaction networks or protein biological information such as gene expression data and gene ontology (GO) knowledge can improve the accuracy and reliability of protein–protein interaction data.

There are different ways to construct a weighted PPI network. For instance, we can calculate the protein similarity according to the topological relationship between proteins to obtain a weighted PPI network. We can also use some biological information, such as GO or gene expression, to calculate the credibility between proteins to get a weighted PPI network. In this paper, we combine the biological information of proteins with the topological information of the protein–protein interaction network to measure the degree of trust between proteins. Then construct a weighted protein–protein relationship network. To calculate the topological similarity between proteins, we introduce the similarity metric *HOCN* proposed by Wang [14] et al., based on the ${Jaccard}^{, }s$ similarity coefficient. The main idea is to estimate the topological similarity metric between nodes based on the high-order public domain of two adjacent nodes. ${Jaccard}^{, }s$ coefficient similarity is a similarity measure proposed by $Jaccard$ et al. The ${Jaccard}^{, }s$ coefficient similarity between two neighbor proteins $v$ and $u$ is defined by Eq. (1):

1 $$JCS\left( {v,u} \right) = \frac{{\left| {CN\left( {v,u} \right)} \right|}}{{\left| {N\left( v \right) \cup N\left( u \right)} \right|}}$$

where $N\left(v\right)$ and $N\left(u\right)$ represent the set of adjacent points of $v$ and $u$ respectively. $N\left(v\right)\cup N\left(u\right)$ represents the union set of adjacent points of $v$ and $u$. $CN\left(v,u\right)$ represents the set of common adjacency points of $v$ and $u$, namely $N\left(v\right)\cap N\left(u\right)$. $\left|N\left(v\right)\cap N\left(u\right)\right|$ and $\left|N\left(v\right)\cup N\left(u\right)\right|$ represent the number of common adjacent points and unions sets of $v$ and $u$, respectively.

*HOCN* is proposed based on the Jaccard similarity coefficient, and its definition is shown in Eq. (2). The topological similarity between protein $v$ and protein $u$ is determined by not only the Jaccard similarity coefficient but also the degree of connection between their common neighborhood and edge ($v,u$). The degree of connection between the common neighborhood and the edge ($v,u$) is defined as $CNS$, as shown in Eq. (4).

2 $$HOCN\left(v,u\right)=\frac{\left(JCS\left(v,u\right)+CNS\left(v,u\right)+\left|CN\left(v,u\right)\right|\right)}{\left(\left|CN\left(v,u\right)\right|+1\right)}$$

3 $${JCS}^{*}=JCS\left(v,w\right)*JCS\left(w,u\right)$$

4 $$CNS\left(v,u\right)=\sum_{w\in CN\left(v,u\right)}\left({JCS}^{*}\right)$$

Gene Ontology GO is one of the most comprehensive ontology databases in bioinformatics. GO provides a series of GO terms to describe the characteristics of gene products, mainly including three aspects: biological process (BP), cell component (CC), and molecular function (MF). If two proteins have more GO terms in common, the more specific information the GO terms describe, and the higher the biological semantic similarity between the two proteins. In this paper, we calculate the biological similarity $sim\left(v,u\right)$ between protein $v$ and $u$ according to the number of GO terms and the number of annotated proteins in GO terms as follow.

5 $$sim\left(v,u\right)=\left|C\left(v,u\right)\right|\times \mathit{log}{\left(\frac{min\left|{S}_{i}\left(v,u\right)\right|}{Smax}\right)}^{2}$$

Protein $v$ and $u$ are both annotated by multiple different GO terms. $C\left(v,u\right)$ represents the GO term set in which protein v and u are annotated by the same GO term. ${S}_{i}\left(v,u\right)\left(1\ll i\ll n\right)$ represents the set of proteins annotated by each GO term in the GO terms shared by proteins $v$ and $u$. $Smax$ represents the maximum number of proteins annotated by a GO term among all GO terms.

To calculate the similarity of two proteins $v$ and $u$, we combine the topological similarity and biological similarity between proteins, and its definition is shown in Eq. (7).

6 $$merge\left(v,u\right)=sim\left(v,u\right)+HOCN\left(v,u\right)$$

7 $$Weight\left(v,u\right)=\sqrt{merge\left(v,u\right)}$$

### Generating supervised features for protein complex prediction

Extracting key features from protein complexes indicates that protein complexes are crucial in our research. So far, a lot of research has been done in this area. We designed 16 features extracted from weighted and unweighted networks to describe protein complexes. A detailed description of the characteristics is shown below.

1. Density: Density is an essential feature in the network and has been widely used in protein complex identification. For an unweighted graph, if $G=\left(V,E\right)$ has $\left|E\right|$ edges, the density is defined as $\left|E\right|$ divided by the theoretical maximum possible number of edges in the graph ${\left|E\right|}_{max}$, ${\left|E\right|}_{max}=\left|V\right|\times \left(\left|V\right|-1\right)/2$. For a weighted graph, set $G=\left(V,E,W\right)$, the weight of the edge $\left(v,u\right)$ is $w\left(v,u\right)$, and its density is defined as shown in formula (8).
  8 $${d}_{w}\left(G\right)=\frac{{\sum }_{u\in V,v\in V}w\left(v,u\right)}{\left|V\right|\times \left(\left|V\right|-1\right)}$$
2. Degree statistics: For unweighted graphs, the node degree is defined as the number of neighbor nodes of the node. For weighted graphs, the node degree is defined as the sum of the weights between the node and its connected nodes. We choose the maximum, average and median of the node degree of the weighted graph and unweighted graph as the sub-graphs features.
3. Edge weight statistics: Edge weight is also an essential feature of weighted networks. It is similar to node degree, and both describe the characteristics of edges in the network. We choose the average and variance of all edge weights in the sub-graphs as the features of the sub-graphs.
4. Degree-related attributes: Degree-related attributes can test the connectivity between a node in the sub-graphs and its neighbor nodes. Each node is defined as the average number of connections of the nearest neighbor nodes of the node, that is, the average degree. We choose the average and variance of the related attributes of the node degree in the sub-graphs as the characteristics of the sub-graphs.
5. Modularity: Modularity indicates the tightness of node connections in the sub-graphs. For a weighted graph $G=\left(V,E,W\right)$, any sub-graph $SG\in G$, let the sum of the weights of the inner edges of $SG$ be ${d}_{w}^{in}\left(SG\right)={\sum }_{u,v\in SG;\left(u,v\right)\in E}w\left(u,v\right)$. The sum of the weights of $SG$ and the external node connecting edge is ${d}_{w}^{out}\left(SG\right)={\sum }_{v\in SG;u\notin SG;\left(u,v\right)\in E}w\left(u,v\right)$. Then the $SG$ modularity ${M}_{SG}$ is defined as shown in formula (9).
  9 $${M}_{SG}=\frac{{d}_{w}^{in}\left(SG\right)}{{d}_{w}^{in}\left(SG\right)+{d}_{w}^{out}\left(SG\right)}$$
6. Clustering coefficient: For unweighted graphs, the clustering coefficient of node *v* is the ratio of the number of triangles to the number of triangles that may be formed. Its definition is shown in the formula (10).
  10 $$C=\frac{2T\left(v\right)}{\left|N\left(v\right)\right|\times \left(\left|N\left(v\right)-1\right|\right)}$$

$T\left(v\right)$ represents the number of triangles passing through node $v$. $N\left(v\right)$ represent the set of adjacent points of node $v$. We choose the variance of the clustering coefficient in the unweighted graph as its clustering coefficient feature. The definition of the clustering coefficient in the weighted graph is shown in formula (11).

11 $$CCW\left(v\right)=\frac{{\sum }_{\left(j,h\right)\in N\left(v\right)}w\left(v,j\right)+w\left(v,h\right)}{w\left(v\right)\left({k}_{v}-1\right)}$$

12 $$w\left(v\right)={\sum }_{j\in N\left(v\right)}w\left(v,j\right)$$

where ${k}_{v}$ represents the number of neighbor nodes of node *v*, and $w\left(v,j\right)$ represents the weight of the edge between nodes $v$ and $j$. $w\left(v\right)$ represents the sum of the weights of the edges between node $v$ and all adjacent nodes. We choose the average and maximum weighted graph clustering coefficient values as their clustering coefficient characteristics.

### The first stage of protein complex candidate recognition

**Fig. 2:**
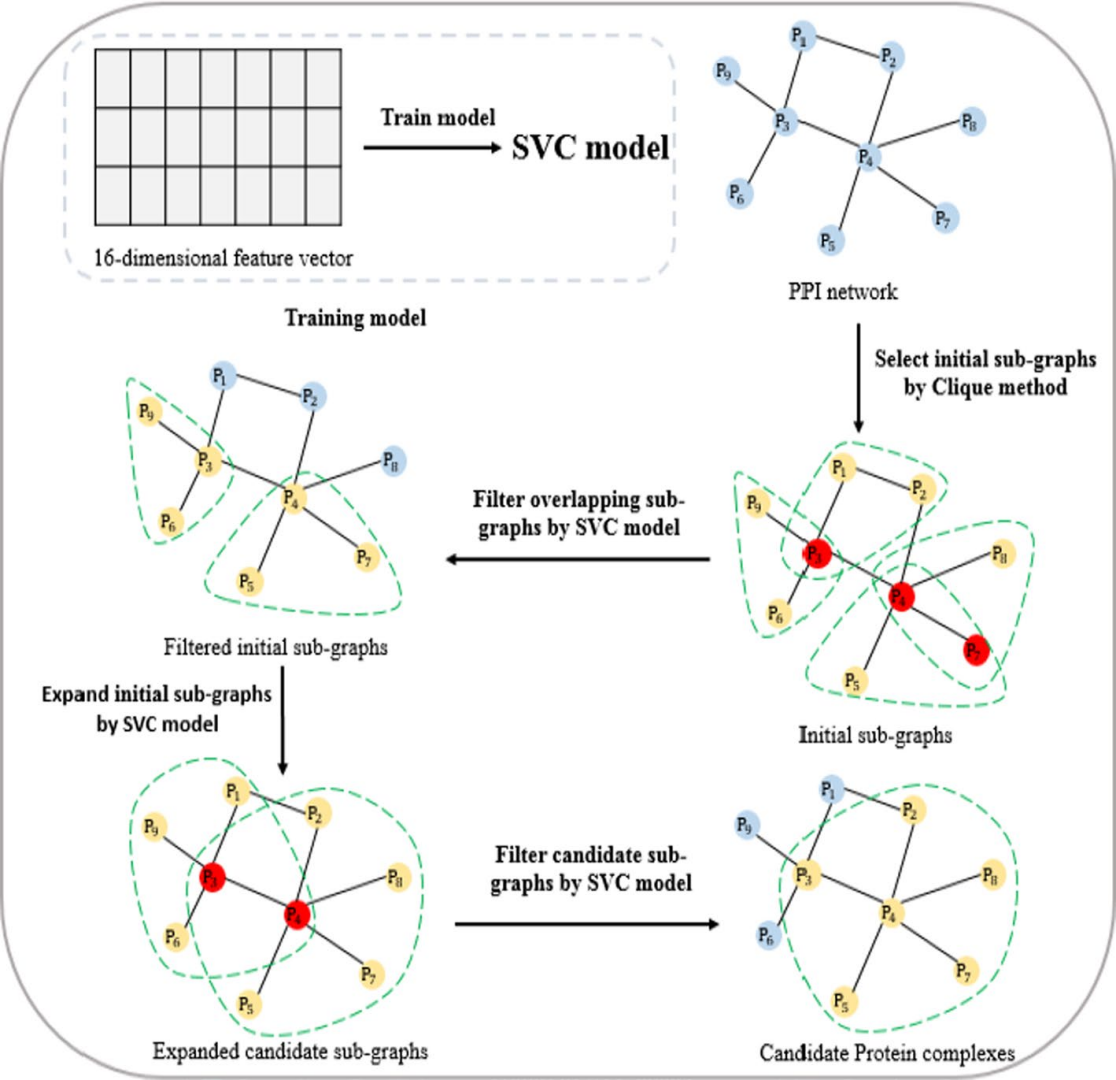
Overall flow chart of SVCC model

This section proposes a supervised learning method SVCC for identifying protein complexes from protein interaction networks. The supervised learning method SVCC mainly includes four steps: (1) the first stage recognition model training; (2) sub-graphs selection; (3) sub-graphs expansion; (4) sub-graphs filtration. The overall flow chart of SVCC is shown in Fig. 2.

#### The first stage recognition model training

In this study, we apply the support vector machine algorithm SVC [26] to predict protein complexes from the protein interaction network. SVC is a support vector machine algorithm mainly used to solve classification problems. The main idea of SVC is to construct an optimal decision hyperplane in the feature space to maximize the distance between the two types of samples closest to the plane on both sides of the plane. Thus, it provides good generalization ability for the classification problem. Compared with other classification methods, SVC requires relatively fewer sample data. Because SVC introduces a kernel function, SVC can easily cope with high-dimensional or nonlinear data samples.

We extract 16 topological features of protein complexes in the network from positive and negative examples, namely the feature vectors of protein complexes. It combines them to obtain the training set. After constructing the training set, we use the training set as input data to train the SVC model. We conducted parameter tuning tests on the main hyperparameters C and Degree in the SVC model. Based on the preliminary experimental results, we chose the hyperparameter C and Degree are 3 and 4 in our experiments, respectively. The various parameters of the SVC model used in this article are shown in Table 1.

**Table 1:** Model parameter settings

Parameters	Value
C	3
Kernel	Poly
Degree	4
Gamma	scale
Coef0	0
Probability	True
Tol	0.001
Cache_size	200

#### Sub-graphs selection

We use the trained SVC model to predict protein complexes from the protein interaction network. We first use the Clique [27] algorithm to search for the largest sub-graph in the protein interaction network. The Clique algorithm is based on the depth-first search algorithm for the largest group in the network. We choose the sub-graph with the number of proteins greater than or equal to 3 as the initial sub-graph. Since the initial sub-images may overlap, we need to filter the initial sub- graphs. We use the trained SVC model to determine the probability that each sub-graph is an actual complex and arrange them in descending order of likelihood. For any sub-graph ${C}_{i}$, calculate the number of overlapping proteins between it and the sub-graph ${C}_{k}$ whose probability is lower than it. If the number of overlapping proteins exceeds the given threshold $\mathrm{\alpha }$, the sub-graph ${C}_{k}$ is filtered out. Repeat the above process to form the final initial set of sub-graphs. For the filtering threshold α, since the number of proteins in the initial subgraph we obtained is greater than or equal to 3, if the threshold α is set to 1, it will lead to too many filtered subgraphs. If the threshold α is greater than 2, many subgraphs with a protein number of 3 cannot be correctly discriminated. Therefore, we set the threshold α = 2. In the sub-graph selection stage, the initial sub-graph structures we obtained by the Clique method are usually relatively simple. Many sub-graph structures contain only 3 proteins. During sub-graph selection, filtering strategy based on the number of proteins in common between the candidate sub-graphs is simple and efficient.

#### Sub-graphs expansion

For any sub-graphs ${C}_{i}$, the set of adjacent points is $N\left({C}_{i}\right)$, and any node $v$ in $N\left({C}_{i}\right)$ is selected to join the subgraph ${C}_{i}$. Then, the trained model is used to determine the probability that $\left\{{C}_{i}\cup v\right\}$ is a true compound, and the node $v$ with the highest probability increase is selected and added to the subgraph ${C}_{i}$. Repeat the above process until there is no node in $N\left({C}_{i}\right)$ so that after it joins ${C}_{i}$, the probability that the subgraph ${C}_{i}$ an actual complex increase. At this point, the subgraph ${C}_{i}$ is expanded to form a candidate sub-graph.

#### Sub-graphs filtration

Candidate sub-graphs may also overlap, so we need to filter the candidate sub-graphs. As in the first step, we use the trained model to determine the probability that the candidate sub-graphs are an actual complex and arrange them in descending order of likelihood. After sub-graph expansion operation, the sub-graph structures become much more complex. It is more appropriate to use the overlap ratio at this stage to determine whether the sub-graphs overlap. For any candidate sub-graphs ${C}_{i}$, we calculate the overlap ratio $overlap\left({C}_{i},{C}_{k}\right)$ between it and the candidate subgraph ${C}_{k}$.

13 $$overlap\left({C}_{i},{C}_{k}\right)=\frac{\left|{C}_{i}\cap {C}_{k}\right|}{\left|{C}_{i}\cup {C}_{k}\right|}$$

If the overlap rate exceeds the set overlap threshold β, we merge the two candidate subgraphs. Otherwise, the candidate sub-graphs ${C}_{k}$ is filtered out. Repeat the above process to obtain candidate protein complexes. We test the optimal value of the overlap threshold β in the interval 0 to 1. Based on the preliminary experiments, we set β as 0.8 in this study.

### The second stage of the final protein complex classification

We apply the supervised learning method SVCC to predict candidate protein complexes from protein interaction networks. However, there are a lot of noisy data in the PPI network, and many complex features only exist in specific networks, which are not universal. In addition, the insufficient number of known complexes leads to uncertainty in the supervised learning model. To solve these problems, we choose the network representation learning method to obtain the vector representation of the protein. Then calculate the protein complex vector based on the protein vector representation as to the feature of the protein complex, and train the random forest model RF based on the feature vector. We use the trained RF model to judge whether the candidate protein complex identified by SVCC is a natural protein complex. At the same time, classify the candidate protein complex to further improve the performance of protein complex recognition.

#### Network representation learning

The network representation learning method automatically learns the distributed representation of nodes based on the adjacency information and network topology. Compared with the traditional method of obtaining the topological characteristics of nodes in the network, the network representation learning method can represent the nodes in the protein–protein interaction network as a low-dimensional vector. It can extract the hidden information in the protein–protein interaction network, including the diversity of the connections between protein nodes. We use node2vec [28] to obtain the vector representation of nodes in the PPI network. Node2vec can automatically learn the vector representation of nodes and maximize network and node structure information retention. Node2vec uses a random walk and alias sampling strategy to obtain the structure information of nodes. In addition, a protein complex is a set of proteins. We calculate the vector of protein complex according to the vector representation of the protein. The calculation method is shown in formula (14).

14 $$complex\left({\varphi }_{1},{\varphi }_{2},\dots ,{\varphi }_{m}\right)=avg{\mathbb{Z}}\left(.,j\right) 0\le j<d$$

where ${\varphi }_{i}\left(i=\mathrm{1,2},\dots ,m\right)$ is the vector representation of protein nodes in a protein complex ${\mathbb{Z}}$ is the matrix composed of the vector representation ${\varphi }_{i}$ of protein nodes in a protein complex, $d$ is the dimension of ${\varphi }_{i}$, and ${\mathbb{Z}}\left(.,j\right)$ is the j-th column in matrix ${\mathbb{Z}}$.

#### Random forest model

The random forest model [20] was used to classify the candidate protein complexes obtained by SVCC. Random forest uses multiple classification trees to distinguish and organize data, and it is a kind of cluster classification model. While classifying the data, it can also give a score of the importance of each variable and evaluate the role of each variable in the classification. The random forest model uses a random method to build a forest. The forest comprises many decision trees, and there is no correlation between each decision tree. When new sample data enters, each decision tree in the random forest is judged separately. For classification problems, voting is usually used. The category with the most votes is used as the final model output. Compared with other classification methods, the random forest can handle high-dimensional data without feature selection. It has good performance for extensive sample data and can also understand variables importance. In addition, the introduction of randomness makes random forests have an excellent anti-noise ability.

#### Candidate protein complex classification

We first obtain the vector representation of the protein in the protein interaction network through the network representation learning method Node2vec. Then calculate the average value of the protein vector representation in the protein complex as the vector of the protein complex. We calculate the vector representation of the protein complex in the positive and negative examples as the feature vector of the protein complex. Subsequently, combine the feature vector of the positive and negative samples to obtain the training set. After constructing the training set, we use the training set as input data to train the RF model. Then, we also use the network representation learning method Node2vec to calculate the feature vector of the candidate protein complex identified by SVCC as the test set. We use the trained RF model to classify the feature vector of the test set. Then, it will be marked as a positive example of the protein the complex is the final predicted protein complex.

## Datasets and evaluation metrics

### Datasets

In this study, we use the human protein interaction network and yeast protein interaction network as experimental data. The human protein interaction network is downloaded from the Human Protein Reference Database (HPRD) [22]. The yeast protein interaction network comes from the extensive yeast data set DIP [21]. For these two kinds of PPI networks, we removed the repetitive and self-connected protein relationships in the network. Finally, we obtained the basic information of the two protein interaction networks, as shown in Table 2. The standard human protein complex data set we use is also downloaded from HPRD, including 1514 human protein complexes. The standard yeast protein complex data set comprises four common yeast standard protein complex data sets: MIPS [29], SGD [30], TAP60 [31], Aloy [32]. It contains 732 yeast protein complexes.

**Table 2:** Basic information of two protein interaction networks

Dataset	Numbers of nodes	Number of edges	Avg numbers of neighbors
DIP	3490	11,189	6.412
HPRD	7307	29,213	7.996

Our training data contains positive examples and negative examples. The positive examples are the standard human protein complex data set and the standard yeast protein complex data set described above. In addition, the standard yeast protein complex data set comprises four common yeast standard protein complex data sets. Therefore, the data set will contain protein molecules that do not exist in the DIP network. The protein complexes predicted from the PPI network will not have protein molecules present in the protein interaction network. Therefore, when experimenting on the DIP network, it is necessary to filter out the protein molecules in the positive protein complex that do not belong to the DIP network. Our negative example is generated by randomly selecting nodes from the PPI network, and its size is consistent with the positive sample. In particular, the number of protein molecules contained in the protein complex in both the positive and negative examples is greater than or equal to 3.

### Evaluation metrics

We used four performance evaluation indexes to evaluate the predicted protein complexes: $precision$,$recall, F-score$,$P-value$.

Suppose that $B=\left\{{b}_{1},{b}_{2},\dots ,{b}_{m}\right\}$ and $P=\left\{{p}_{1},{p}_{2},\dots ,{p}_{n}\right\}$ represent the standard protein complex set and the predicted protein complex set, respectively. If selecting a real protein complex $b\in B$ and a predicted protein complex $p\in P$, we can calculate their similarity, namely neighborhood affinity score $NA$ as Eq. (15).

15 $$NA\left(b,p\right)=\frac{{\left|{V}_{b}\cap {V}_{p}\right|}^{2}}{{V}_{b}\times {V}_{p}}$$

where ${V}_{b}$ and ${V}_{p}$ represent the collection of protein rmolecules in complexes $b$ and $p$, respectively. $\left|{V}_{b}\cap {V}_{p}\right|$ represents the number of proteins shared in the two protein complexes.

Generally speaking, if $NA\left(b,p\right)$> 0.25, the two protein complexes are considered to be matched. Let P and B denote the set of predicted protein complexes and standard protein complexes, respectively. Let ${N}_{cb}$ denote the number of standard protein complexes that match at least one predicted protein complex. ${N}_{cp}$ denote the number of predicted protein complexes that match at least one standard protein complex. Then the definition of $precision$ and $recall$ are shown as Eq. (16) and (17).

16 $$precision=\frac{{N}_{cp}}{\left|P\right|}$$

17 $$recall=\frac{{N}_{cb}}{\left|B\right|}$$

$F-score$ is defined as the harmonic average of $precision$ and $recall$, that is, a reasonable mixture of $precision$ and $recall$, and its definition is shown as Eq. (18).

18 $$F-score=\frac{2\times precision\times recall}{precision+recall}$$

In addition, in this article, we also use the biological process annotations in the gene ontology to analyze the biometric significance of protein complexes identified by different methods. The biological statistical significance of a protein complex can be marked by its biological function. Calculated by hypergeometric distribution, the definition is shown as Eq. (19).

19 $$P-value=1-\sum_{i=0}^{k-1}\frac{\left(\genfrac{}{}{0pt}{}{\left|F\right|}{i}\right)\left(\genfrac{}{}{0pt}{}{\left|V\right|-\left|F\right|}{\left|C\right|-i}\right)}{\left(\genfrac{}{}{0pt}{}{\left|V\right|}{\left|C\right|}\right)}$$

where $\left|V\right|$ represents the number of protein nodes of the corresponding entire species. $C$ represents the predicted protein complex, which contains $k$ proteins and is annotated by the gene ontology functional group $F$. The smaller the $P-value$ of a protein complex is, the more likely it is to be annotated with the same function, and the more likely it is to be a true complex.

Our proposed method is based on supervised learning. To evaluate our method using an independent testing set, we follow the previous work to evaluate the proposed method in a five-fold cross-validation experimental setting.

## Results and discussion

This section introduces our comparative experiment in detail, mainly composed of three parts. In the first part, we compare the performance of our method with several existing protein complex prediction methods. The second part analyzes the impact of different factors on the experimental performance, including classification models, network representation learning methods, and feature sets. In the third part, the biological significance of the predicted complexes is evaluated and discussed.

### Comparison results with other methods

To validate the effectiveness of our method in predicting protein complexes, we compared our method with MCODE [2], COACH [6], CMC [3], ClusterONE [8], GANE [15], EWCA [14], SLPC [16], and SVCC only on two protein interaction networks of DIP and HPRD. To compare these methods as fair as possible, we use a five-fold-cross-validation experimental setting to identify protein complexes. We divided the standard set of protein complexes for DIP and HPRD into five parts as $\left\{{C}_{1},{C}_{2},{C}_{3},{C}_{4},{C}_{5}\right\}$. In each crossover experiment, we use 4 of them as the training set and train the SVC model and the random forest model to recognize the complexes in the network. Since the identified complexes may contain the complexes of the training set, we remove the complexes that overlap with the training set to obtain ${R}_{1}$, where the overlap threshold is set to 0.9, calculated by the formula (13). After five rounds of such experiments, we combined the set of five complexes identified as $\{{R}_{1},{R}_{2},{R}_{3},{R}_{4},{R}_{5}\}$. And remove the complexes in which the overlap ratio is greater than 0.6. The remaining protein complexes are then taken as the final result and evaluated using a standard collection of protein complexes. The MCODE and ClusterONE methods are processed by Cytoscape [33]. The parameters of the other methods are set according to their authors' recommendations. Our method used node2vec to learn the vector representation of proteins on the protein interaction network. The parameters of node2vec set to q = 1, *p* = 8, dimensions = 64. The comparison results between our method and other methods are shown in Table 3.

**Table 3:** Comparison of experimental results on DIP and HPRD data sets

Dataset	Method	Number	Precision	Recall	F-score
DIP	MCODE	72	0.5138	0.1010	0.1689
	COACH	747	0.4310	0.4685	0.4490
	CMC	709	0.3004	0.4631	0.3644
	ClusterONE	363	0.5041	0.3661	0.4241
	GANE	326	0.6012	0.4303	0.5016
	EWCA	1028	0.5642	0.4767	0.5168
	SLPC	766	0.6057	0.4631	0.5249
	SVCC only	946	0.5898	0.4699	0.5231
	Our	514	0.7684	0.4330	**0.5539**
HPRD	COACH	1914	0.3322	0.5805	0.4226
	CMC	2399	0.3384	0.7741	0.4710
	ClusterONE	875	0.3942	0.3348	0.3621
	GANE	755	0.3668	0.3124	0.3374
	EWCA	1915	0.4976	0.5832	0.5370
	SLPC	2431	0.4452	0.6901	0.5412
	MCODE	137	0.5182	0.1050	0.1746
	SVCC only	2649	0.3812	0.8243	0.5213
	Our	1252	0.5559	0.7186	**0.6268**

The highest F-score is in bold

Table 3 shows the results of our method compared with other methods on the yeast PPI network DIP and the human PPI network HPRD. When we use the yeast PPI network DIP as the experimental network, our method achieves the highest F-score of 0.5539, which is much higher than the unsupervised learning methods. At the same time, the F-score obtained by using only the SVCC method is 0.5231, which is slightly lower than the F-score of 0.5249 of the supervised learning method SLPC. After using the RF model to classify the candidate protein complexes identified by SVCC, the experimental performance improved by 3%. Using the human PPI network HPRD as the experimental network, our method achieves the highest F-score of 0.6268. Compared with unsupervised learning methods, our method improves by at least 15%, except for EWCA. At the same time, it is also an increase of nearly 10% compared with EWCA. Compared with the supervised learning method SLPC, our method improves by about 8%. When we only use the SVCC.

method, the obtained F-score is 0.5213. After using the RF model to classify the candidate protein complexes identified by SVCC, the experimental performance improved by about 10%. It can be seen from the above experiment that using a trained RF model to classify candidate protein complexes predicted by SVCC can significantly improve the performance of the experiment. In summary, our method achieves good performance on both the yeast PPI network and the human PPI network. Especially in the human PPI network, our method is significantly better than other methods. Therefore, our method is superior to the existing protein complex prediction methods.

We also note that the precision is much higher than recall on DIP network, but vice-versa on HPRD network. This maybe because the scale of HPRD is much larger than that of DIP (as seen in Table 2). It makes some methods can identify a large number of complexes on HPRD. We have supplemented the number of complexes identified in Table 3. From Table 3, we can see that the six methods of COACH, CMC, EWCA, SLPC, SVCC and our method can identify more than 1000 protein complexes on the HPRD network, which is much higher than the number of standard protein complexes, namely $\left|P\right|\gg \left|B\right|$ in Eqs. 16 and 17. This leads to the precision is less than the recall on HPRD network.

### Comparison with other classification models

We use the RF model to classify the candidate protein complexes obtained by the SVCC method. The above experiments show that the RF model can significantly improve the experimental performance. To further verify the effectiveness of the random forest model RF, we also chose to train other supervised learning models to classify candidate protein complexes.

**Fig. 3:**
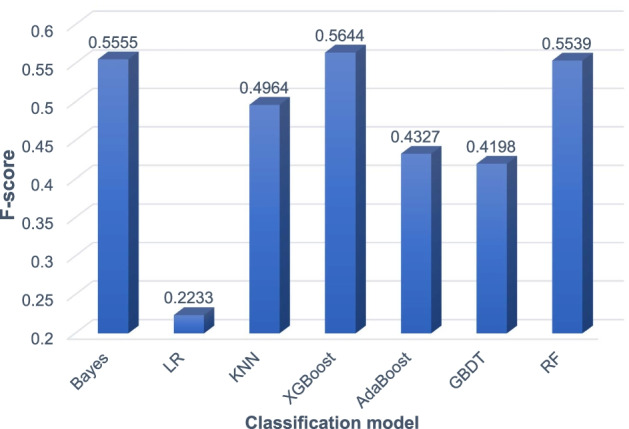
Experimental comparison results of supervised models on the DIP network

**Fig. 4:**
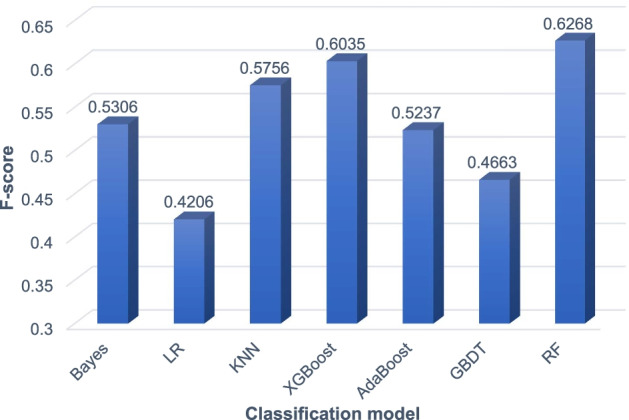
Experimental comparison results of superrvised models on the HPRD network

We trained naive Bayes (Bayes), logistic regression (LR), KNN, XGBoost, AdaBoost, and gradient boosted tree GBDT six supervised learning models and compared their experimental results with the random forest model RF. We conducted parameter tuning experiments on these supervised learning models. We selected the best test parameters which are shown in Table 4. The results of comparing the RF model and the other six supervised learning models on the yeast PPI network DIP and the human PPI network HPRD are shown in Figs. 3 and 4.

**Table 4:** Parameter settings of six supervised learning models

ID	Model	Parameters
1	RF	n_estimators = 1000
2	LR	C = 1.0
3	KNN	n_neighbors = 5
4	XGBoost	booster = gbtree, learning_rate = 0.3, max_depth = 6, min_child_weight = 1
5	AdaBoost	base_estimator = DecisionTreeClassifier, algorithm = SAMME, n_estimators = 350, learning_rate = 0.4
6	GBDT	learning_rate = 0.1, n_estimators = 100, max_depth = 2, min_samples_split = 1.0, min_samples_leaf = 2

It can be seen from Fig. 3 that on the yeast PPI network DIP, the XGBoost model achieves the highest F-score of 0.5644. Bayes and RF achieve the second and third highest F-scores, 0.5555 and 0.5539, respectively. The XGBoost model is about 1% higher than the RF model. It can be seen from Fig. 4 that on the human PPI network HPRD, the RF model achieves the highest F-score of 0.6268. The XGBoost model also achieved a high F-score of 0.6035. But the Bayes model only achieved an F-score of 0.5036. In summary, the RF model achieves the best performance on the human PPI network HPRD and good performance on the yeast PPI network DIP. Therefore, we finally choose the RF model to classify the candidate protein complexes obtained by the SVCC method.

### Influence of different network representation learning methods

**Fig. 5:**
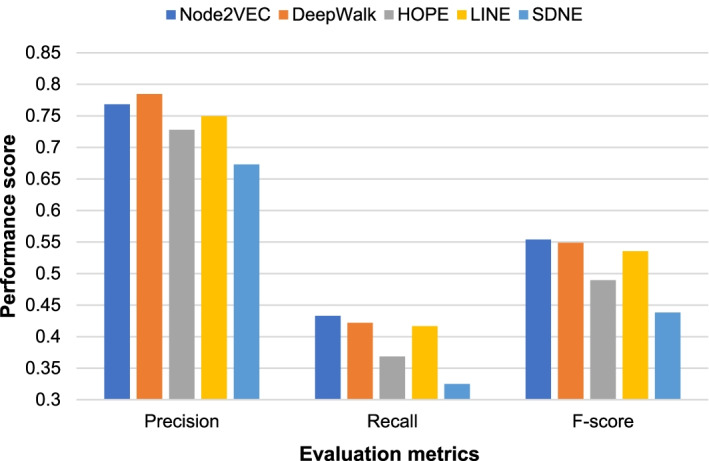
The impact of different network representation learning methods on the experimental performance of the DIP network

**Fig. 6:**
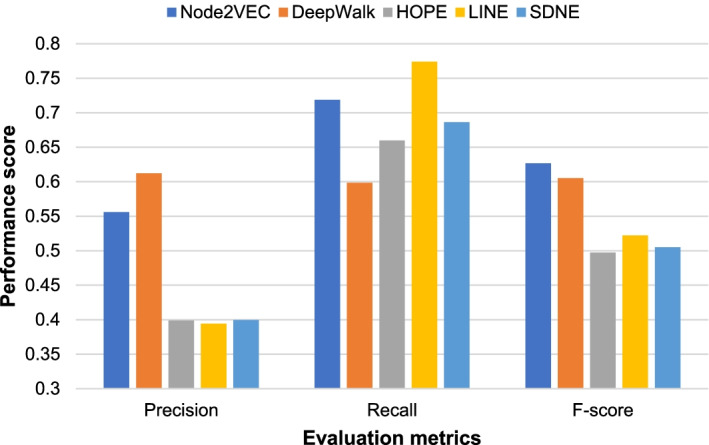
The impact of different network representation learning methods on the experimental performance of the HPRD network

In this study, we applied the network representation learning method node2vec to obtain the vector representation of the protein. Then, according to the protein vector representation, the protein complex vector is calculated as the feature of the protein complex. Finally, the candidate protein complexes are classified through the feature vector training model. To verify the effect of node2vec for our method, we also evaluate four other network representation learning methods, including DeepWalk [13], HOPE [34], LINE [35], and SDNE [36]. Most of the parameters of these five network representation learning methods are set to default values, and only a few parameters with significant influence are tested (such as dimension, etc.). The specific parameter settings of the five network representation learning methods are shown in Table 5. The comparison between node2vec and the other four network representation learning methods on the yeast PPI network DIP and the human PPI network HPRD is shown in Figs. 5 and 6.

**Table 5:** Parameter settings of five network representation learning methods

ID	Method	Parameters
1	Node2VEC	walk-length = 80, number-walks = 10, p = 8.0, q = 1.0, dimensions = 64
2	DeepWalk	walk-length = 80, number-walks = 10, dimensions = 64
3	HOPE	dimensions = 64
4	LINE	epoch = 5, order = 3, clf-ratio = 0.5, dimensions = 64
5	SDNE	alpha = 1e-6, beta = 5, nu1 = 1e-5, nu2 = 1e-4, batch_size = 200, epoch = 5, learning_rate = 0.01, dimensions = 64

As shown in Figs. 5 and 6, our method achieves higher F-scores than other network representaion learning methods on both the yeast PPI network DIP and the human PPI network HPRD when using the Node2vec method to obtain the vector representation of the protein. It can be seen that on learning methods on both the yeast PPI network DIP and the human PPI network HPRD when using the Node2vec method to obtain the vector representation of the protein. It can be seen that Node2vec achieves the highest F-score on both DIP and HPRD networks. We also note that Deep Walk outperforms Node2vec in precision, especially for the HPRD network. DeepWalk randomly and uniformly selects nodes in the network during random walks [13]. Node2vec uses two parameters p and q to control the direction of the sampling during random walks [28]. In other words, Node2vec uses a flexible and biased random walk sampling strategy to trade off the local and global structure of the network. Compared to DIP network, the scale of HPRD network is much larger. From Fig. 6, the results suggest that Deepwalk can achieve higher precision on larger networks such as HPRD network. But Node2vec can achieve higher recall and F-score than Deepwalk. Overall, Node2vec achieve the best performance among the five methods on both DIP and HPRD networks.

### The impact between binary and multiple classification models

The training set data we selected when training the SVC model and the RF model is the same, including positive and negative examples. The positive data is a standard protein complex data set. The negative data is generated by randomly selecting nodes from the PPI network according to the ratio of the standard protein complexes. Many researchers used multiple classification labels to train supervised learning models in the existing research on protein complex prediction based on supervised learning. To verify the effectiveness of the two-class training set data selected, we also used the three-class training set data to train the supervised learning model for experiments on the DIP and HPRD protein interaction relationship network. The three-category training set data we choose mainly consists of three parts: positive examples, intermediate examples, and negative examples. Among them, the positive and negative data are the same as the binary classification. For the intermediate data, we chose to use the COACH algorithm to identify protein complexes from the PPI network. This is mainly because the possibility of the complexes identified by COACH being real complexes is higher than that of negative sample data but lower than that of positive sample data, which can effectively increase the richness of training sample data. And in order to ensure the accuracy of the experiment, the protein complexes matching the positive example were filtered out. The results of the experimental comparison of the two-class and three-class data on the yeast PPI network DIP and the human PPI network HPRD are shown in Table 6.

**Table 6:** Comparison of experimental results using different classification labels on the DIP and HPRD datasets

Dataset	SVC train set	RF train set	Precision	Recall	F-score
DIP	Two categories	Two categories	0.7684	0.4330	0.5539
		Three categories	0.8726	0.4112	**0.5589**
	Three categories	Two categories	0.5562	0.4467	0.4955
		Three categories	0.5579	0.4494	0.4978
HPRD	Two categories	Two categories	0.5559	0.7186	**0.6268**
		Three categories	0.7891	0.4966	0.6096
	Three categories	Two categories	0.4485	0.4299	0.4390
		Three categories	0.6472	0.2919	0.4023

The highest F-score is in bold

As shown in Table 6, on the yeast PPI network DIP and the human PPI network HPRD, the experimental performance when we train the SVC model with the binary training set data is much better than the three-category training set data. Therefore, we select the binary classification training set data to train the SVC model. When training the RF model on the yeast PPI network DIP, the F-score obtained by selecting the binary training set data is 0.5539. The F-score obtained from the three-class training set data is 0.5589, the difference is less than 1%. When training the RF model on the human PPI network HPRD, the F- score obtained from the two-category training set data is 0.6268. The F-score obtained from the three-category training set data is 0.6096. The performance of the two-category data is about 2% higher than that of the three-category data. Therefore, we select the two-category training set data for the RF model. In summary, to obtain good experimental performance, we choose the two-class training set data when training the SVC and RF models.

### The biological significance of predicted protein complexes

In this section we validate the biological significance of protein complexes based on Gene Ontology GO. In previous complex identification methods, many researchers used $P-value$ to evaluate the biological significance of protein complexes. The $P-value$ indicates the possibility of co-occurring proteins having a common function. If the identified protein complex has a lower $P-value$, it indicates that the co-occurrence of proteins in the complex is not accidental. The lower the $P-value$, the higher the biological significance of the complex, and the more likely it is a significant complex. This paper uses GO term enrichment analysis to determine whether members of a predicted complex have a likely common function. We used LAGO [37] to calculate P-values for protein complexes for functional enrichment analysis and set all parameters in LAGO to default values. LAGO is a fast tool improved based on GO Term Finder [37], which can find important GO terms in the gene name list and calculate the $P-value$ through hypergeometric distribution. Some complexes could have low p-values for multiple different GO terms. In this paper, we chose the best (lowest p-value) GO terms for each complex.

Tables 7 and 8 present the ten protein complexes with lower $P-value$ that we identified on both the DIP and HPRD PPI networks. Moreover, these protein complexes have a high degree of matching with standard protein complexes (calculated by the formula (13)), suggesting that those with low $P-value$ are likely to be genuine protein complexes.

**Table 7:** Ten predicted complexes with low $P-value$ that match the true complexes on the DIP network

ID	Complex	Match	$P-value$		
			GO_Process	GO_Function	GO_Component
1	**YLR148W YAL002W YLR396C YPL045W YMR231W**	1.0	5.73543e-12	7.89663e-08	1.33543e-16
2	**YNL262W YDR121W YBR278W YPR175W**	0.8	6.51289e-12	4.55447e-14	6.52337e-10
3	**YOL094C YBR087W YNL290W YJR068W YMR078C YHR191C**	0.86	6.7513e-11	4.80533e-15	1.4919e-19
4	**YBR123C YOR110W** YGR047C **YAL001C YDR362C**	0.83	3.56116e-16	1.33543e-16	3.81553e-17
5	**YPR162C YBR060C YNL261W YHR118C YML065W** YJL194W **YLL004W**	0.85	2.62478e-19	6.57407e-17	5.95926e-20
6	**YJR043C YJR006W YDL102W**	1.0	9.13462e-10	2.69145e-09	6.52473e-11
7	**YIL033C YJL164C YPL203W YKL166C**	1.0	3.00595e-12	6.52473e-11	9.10894e-15
8	**YPL210C YDL092W** YKL122C **YPR088C YML105C**	0.8	1.29404e-13	1.59353e-12	3.56116e-16
9	**YJL074C YFL008W YDL003W** YER147C **YIL026C YDR180W**	0.83	2.79506e-14	5.83637e-06	2.0486e-12
10	**YPR018W YML102W YBR195C**	1.0	5.70914e-10	5.58191e-07	6.52473e-11

**Table 8:** Ten predicted complexes with low $P-value$ that match the true complexes on the HPRD network

ID	Complex	Match	$P-value$		
			GO_Process	GO_Function	GO_Component
1	**EDC3 DCP1B DDX6 DCP1A EDC4**	0.83	1.16739e-14	3.054e-07	2.53167e-12
2	**HDAC1 RELA NCOR2 HDAC3 PML**	1.0	1.52359e-07	6.80376e-09	5.39083e-07
3	**MED25 MED1 MED9 MED8 MED6** CDK8	0.86	4.48623e-13	1.51728e-07	4.56648e-17
4	**POLR2D POLR2H POLR2A POLR2G POLR2C POLR2E**	0.83	6.12908e-06	7.33099e-11	5.9544e-20
5	**COPS8 COPS5 COPS6 COPS4 COPS2** CUL5 **COPS3**	0.61	1.74854e-20	1.60322e-06	4.16646e-19
6	**JUP BTRC CTNNB1 AXIN1**	1.0	2.55384e-08	3.37375e-07	1.2821e-07
7	**CASP1 CASP2 NLRP1 NOD1**	1.0	2.46197e-10	2.09624e-09	1.83324e-07
8	**FANCE FANCG** HES1 **FANCA FANCF**	0.8	7.87032e-11	6.84817e-06	7.78789e-13
9	**STX3** VAMP7 **SNAP29**	0.83	7.34722e-08	5.99134e-09	1.42065e-08
10	**EED YY1 HDAC2**	1.0	2.7759e-05	2.59935e-05	4.61418e-09

For these complexes with low $P-value$ and high matches to standard protein complexes, we further analyzed them from a biological perspective. In Tables 7 and 8, the proteins that match to standard protein complexes are marked in bold. For examples, complex No.3 in Table 7 overlaps "RFC3—Subunit of heteropentameric Replication factor C": {YNL290W, YOL094C, YBR087W, YMR078C, YHR191C, YCL016C, YJR068W} by 86%. Complex No.5 in Table 8 highly matches "COP9 signalosome complex"[38]: {GPS1, COPS1, COPS2, COPS3, COPS4, COPS5, COPS6, COPS7A, COPS7B, COPS8} by 61%. COP9 signalosome complex plays a critical role in the DNA double-strand break reaction and an ATM target [38]. It regulates the activity of the cullin loop ubiquitin ligase complex by removing ubiquitin proteins from the protein "cullin" scaffold. Also, complex No.8 in Table 8 overlaps "Nucleic and Chormatin Fanconi complex"[39]: {FANCA, FANCC, FANCD2, FANCE, FANCF, FANCG} by 80%. Nucleic and Chormatin Fanconi complex is involved in subcellular localization and functions such as cellular rescue, defense, and virality. The analysis of the predicted complexes suggests our method can effectively predict and identify the meaningful protein complexes from the PPI network.

We also found that some predicted complexes did not match the standard protein complexes, but they still had very low $P-value$, as shown in Table 9. We also marked the proteins that match to standard protein complexes in bold. For example, complex No. 2 on the DIP network in Table 9 overlaps only 25% with “ISWI”: {YBR245C, YFR013W}. These complexes are also of high biological interest, because there may be some undiscovered true protein complexes. These complexes may help biologists looking to identify new protein complexes.

**Table 9:** Eight predicted complexes with low $P-value$ that don’t match the true complexes on the DIP and HPRD networks

Dataset	ID	Complex	Match	$P-value$		
				GO_Process	GO_Function	GO_Component
dDIP	1	**YER082C** YLR222C YCR057C **YLR129W**	0.21	4.76944e-10	3.56521e-05	2.2827e-09
	2	YOR304W **YBR245C** YER164W	0.25	4.24108e-08	2.88883e-08	7.01017e-07
	3	**YJL069C YCR057C YGR090W YER082C** YDR449C	0.33	7.42936e-10	5.70436e-09	2.91387e-11
	4	YHL006C YDR078C YIL132C YLR376C	0.0	3.00595e-12	6.54104e-05	4.55447e-14
HPRD	1	SHC1 **EPOR** CSF2RB JAK2 LYN PTPN6	0.12	1.00517e-08	1.14531e-06	3.6605e-07
	2	**GTF2E2 GTF2B GTF2E1 GTF2F2** TBP	0.2	3.43893e-12	1.97181e-14	1.63409e-13
	3	**MCM10** CDC7 **MCM2** CDKN2A	0.1	2.01548e-06	3.66353e-06	1.16493e-05
	4	CDH18 CDH9 CDH6	0.0	9.51598e-09	4.37102e-06	3.82459e-09

## Conclusions

We proposed a supervised protein complex prediction method with network representation and gene ontology knowledge. We weight the PPI network through the GO knowledge and topological information. Then we extract the topological information of the protein complex according to the weighted PPI network and the unweighted PPI network as its features and construct a training set. Through the constructed training set, the SVCC model is obtained. The SVCC model is used to predict candidate protein complexes from the protein interaction relationship network. Finally, we use the network representation learning method node2vec to obtain the vector representation of the protein complex and train the RF model. The RF model is used to classify the candidate protein complexes. The candidate protein complexes marked as positive by the RF model are the final predicted protein complexes. We evaluate the experimental performance of our method and seven other existing methods on the yeast PPI network DIP and the human PPI network HPRD. The experimental results show that our method effectively detects protein complexes from the PPI network compared with the existing protein complex identification methods. We also analyzed the biological significance of protein complexes predicted by different methods. The results show that the protein complexes predicted by our method have higher biological significance.

Since GO term simlilarity is used in training SVC model in our method, using GO terms to evaluate the predicted complexes has certain limitations. As a future study, we will explore another way than GO terms to evaluate the biological significance of protein complexs.

## Data Availability

The DIP dataset is available at https://dip.doe-mbi.ucla.edu/dip/Main.cgi. The HPRD dataset is available at http://www.hprd.org. The Gene Ontology is available at http://geneontology.org.
